# Adipose mesenchymal stem cells combined with platelet-rich plasma accelerate diabetic wound healing by modulating the Notch pathway

**DOI:** 10.1186/s13287-021-02454-y

**Published:** 2021-07-13

**Authors:** Nesrine Ebrahim, Arigue A. Dessouky, Ola Mostafa, Amira Hassouna, Mohamed M. Yousef, Yasmin Seleem, Eman Abd El Aziz M. El Gebaly, Mona M. Allam, Ayman Samir Farid, Bayan A. Saffaf, Dina Sabry, Ahmed Nawar, Ahmed A. Shoulah, Ahmed H. Khalil, Sami F. Abdalla, Mohamed El-Sherbiny, Nehal M. Elsherbiny, Rabab F. Salim

**Affiliations:** 1 Department of Histology and Cell Biology Faculty of Medicine, Benha University, Benha, Egypt; 2 Stem Cell Unit, Faculty of Medicine, Benha University, Benha, Egypt; 3 Department of Histology and Cell Biology, Faculty of Medicine, Zagazig University, Zagazig, Egypt; 4 School of Interprofessional Health Studies, Faculty of Health and Environmental Sciences, AUT University, Auckland, New Zealand; 5 Department of Clinical Pharmacology, Faculty of Medicine, Benha University, Benha, Egypt; 6 Department of Medical Physiology, Faculty of Medicine, Benha University, Benha, Egypt; 7 Department of Clinical Pathology, Faculty of Veterinary Medicine, Benha University, Moshtohor, Toukh, Qalyubia 13736 Egypt; 8 Department of Pharmacology, Faculty of Pharmacy, Future University, New Cairo, Egypt; 9 Department of Medical Biochemistry and Molecular Biology, Faculty of Medicine, Cairo University, Cairo, Egypt; 10 Department of Medical Biochemistry and Molecular Biology, Faculty of Medicine, Badr University, Cairo, 11562 Egypt; 11 Department of General Surgery, Faculty of Medicine, Benha University, Benha, Egypt; 12 Department of Surgery, & Radiology Faculty of Veterinary Medicine, Benha University, Benha, Egypt; 13 Clinical Department, College of Medicine, AlMaarefa University, Riyadh, Saudi Arabia; 14 Department of Basic Medical Sciences, College of Medicine, AlMaarefa University, Riyadh, Saudi Arabia; 15 Department of Anatomy, Faculty of Medicine, Mansoura University, Mansoura, Egypt; 16 Department of Biochemistry, Faculty of Pharmacy, Mansoura University, Mansoura, 35516 Egypt; 17 Department of Pharmaceutical Chemistry, Faculty of Pharmacy, University of Tabuk, Tabuk, Saudi Arabia; 18 Department of Medical Biochemistry and Molecular Biology, Faculty of Medicine, Benha University, Benha, Egypt

**Keywords:** Diabetic wound, Adipose mesenchymal stem cells, PRP, Notch pathway

## Abstract

**Background:**

Diabetic foot ulceration is a serious chronic complication of diabetes mellitus characterized by high disability, mortality, and morbidity. Platelet-rich plasma (PRP) has been widely used for diabetic wound healing due to its high content of growth factors. However, its application is limited due to the rapid degradation of growth factors. The present study aimed to evaluate the efficacy of combined adipose-derived mesenchymal stem cells (ADSCs) and PRP therapy in promoting diabetic wound healing in relation to the Notch signaling pathway.

**Methods:**

Albino rats were allocated into 6 groups [control (unwounded), sham (wounded but non-diabetic), diabetic, PRP-treated, ADSC-treated, and PRP+ADSCs-treated groups]. The effect of individual and combined therapy was evaluated by assessing wound closure rate, epidermal thickness, dermal collagen, and angiogenesis. Moreover, gene and protein expression of key elements of the Notch signaling pathway (Notch1, Delta-like canonical Notch ligand 4 (DLL4), Hairy Enhancer of Split-1 (Hes1), Hey1, Jagged-1), gene expression of angiogenic marker (vascular endothelial growth factor and stromal cell-derived factor 1) and epidermal stem cells (EPSCs) related gene (ß1 Integrin) were assessed.

**Results:**

Our data showed better wound healing of PRP+ADSCs compared to their individual use after 7 and 14 days as the combined therapy caused reepithelialization and granulation tissue formation with a marked increase in area percentage of collagen, epidermal thickness, and angiogenesis. Moreover, Notch signaling was significantly downregulated, and EPSC proliferation and recruitment were enhanced compared to other treated groups and diabetic groups.

**Conclusions:**

These data demonstrated that PRP and ADSCs combined therapy significantly accelerated healing of diabetic wounds induced experimentally in rats via modulating the Notch pathway, promoting angiogenesis and EPSC proliferation.

## Introduction

Diabetes mellitus (DM) is a worldwide health problem affecting approximately 9.3% of the global population with its prevalence expected to rise by 25% in 2030 and 51% in 2045 [1]. Diabetic foot ulceration (DFU) is one of the most common chronic diabetic complications leading to significant medical, economic, and social burdens. It is estimated that every 30 s, a complicated diabetic lower limb is lost worldwide as 15 to 25% of diabetic patients have a risk to develop a foot ulcer throughout their whole lifetime [2].

The strong regenerative and healing capabilities of skin are intimately linked to the existence of skin stem cells. The epidermal stem cells (EPSC) are a type of autologous adult stem cells that exist in the epidermis and the hair follicles ensuring the preservation of adult skin hemostasis and hair regeneration; moreover, EPSC participates in the repair of the injured epidermis [3]. Typically, the remaining skin stem cells upon the wound surface are linked to a faster curing rate and particularly less scar formation. Normal cutaneous tissue has a diversity of stem cells with multipotent potentiality leading to, theoretically, any wound can easily rely on skin stem cells to reach physiological repair. Nevertheless, in profound skin wounds, the remaining skin stem cells cannot experience normal differentiation capacity and complete the anatomical structure and functional skin repair according to the preprogrammed pathways. Therefore, the healing progression may be disrupted, ultimately developing scar tissue devoid of the hair follicle and sweat glands [4]. This indicates that the process of wound healing is associated with interaction among cells, complex regulation of the extracellular matrix, and various paracrine elements [5].

Provided the complexity of the multifactorial and multicellular processes of wound healing, it is believable that a therapeutic approach targeting different signaling pathways that control cellular processes significant for wound healing would likely serve as a considerable solution for DFU therapy. The Notch signaling pathway is an evolutionarily preserved signaling mechanism with an extremely pleiotropic action. Notch signaling is essential for cell fate determination. Besides, it also plays a critical role in regulating proliferation, angiogenesis, and apoptosis/survival, processes that are intensely disturbed in diabetic wounds [6]. Notch signaling is actually a cell–cell interaction mechanism triggered as a result of the interaction between membrane-bound Notch receptors (Notch 1–4) and their particular ligands (Delta-like 1, 3, 4, and Jagged 1–2) on juxtaposed cells. This interaction induces γ-secretase-mediated cleavage and translocation of the Notch intracellular domain (ICD) into the nucleus, where it forms a transcriptional activation complex inducing the expression of downstream target genes, such as Hairy Enhancer of Split-1 (Hes1) and Hey1 [7]. This signaling pathway performs essential tasks during development and throughout the regulation of adult tissue homeostasis. Besides, it plays a vital role in the postnatal physiology of the skin as well as in normal wound healing through the positive regulation of cell migration, angiogenesis, and inflammation [8].

Several innovative treatment options intended for DFU management have been discovered, like bioengineered skin substitutes, negative pressure dressings, and hyperbaric oxygen therapy. Nevertheless, the typically effective therapies remain inadequate. Therefore, it is essential to use more successful and efficient therapies like the use of autologous biologics, such as mesenchymal stem cell (MSC)-based therapies and platelet-rich plasma (PRP), which hold considerable promise to improve tissue regeneration in addition to chronic wound care management strategies [9]. PRP can be acquired throughout an autologous manner, via centrifugation of the patient’s blood leading to a plasma fraction with a platelet concentration greater than that of the circulating blood. Indeed, autologous therapies using PRP need meticulous preparation [10]. The therapeutic properties of PRP are mostly endorsed to the release of platelet growth factors after its activation. These groups of growth factors include epidermal growth factor (EGF), platelet-derived growth factor (PDGF), fibroblast growth factor (FGF), vascular endothelial growth factor (VEGF), and insulin-like growth factor (IGF-1, IGF2) which are recognized to favor tissue regeneration [11]. On the other hand, adipose-derived MSCs are adult multipotent stem cells with self-renewal potentiality, which can differentiate directly into different lineages and secrete paracrine elements starting the process of tissue regeneration. The plentiful supply of the adipose tissue, simple isolation procedure, wide proliferative capabilities ex vivo, besides their capacity to secrete pro-angiogenic growth factors made them an ideal cell type to be used as a new therapy for the treatment of non-healed wounds. Moreover, the MSC secretome initiates healing simply by inducing a shift from pro-inflammatory to anti-inflammatory cytokine production at the injury site [12]. Of note, recent studies reported new potential application of adipose-derived MSCs in the treatment of COVID-19 [13–15].

In the present study, we aimed to evaluate the therapeutic efficiency of PRP and ADSCs both individually and in combination in diabetic wound healing. Furthermore, we investigated the role of the Notch signaling pathway in controlling wound healing.

## Materials and methods

### Experimental animals

Adult male albino rats (180–200 g), 6 weeks of age, were purchased from the Experimental Animal Unit, Faculty of Veterinary Medicine, Benha University, Egypt. The rats were bred and maintained in an air-conditioned animal house under specific pathogen-free conditions. All animals were housed in clean cages and given a standard diet and clean water ad libitum. The rats were subjected to a normal light/dark cycle (12-h light-dark cycle starting at 8:00 AM) and room temperature (23 ± 3°C and allowed free access to chow and water. This study was carried out in strict accordance with the recommendations in the Guide for the Care and Use of Laboratory Animals of the National Institutes of Health (NIH publication No. 85–23, revised 2011). All protocols were approved by the institutional review board for animal experiments of the Faculty of Medicine, Benha University, Egypt (BUFM 3 January 2018).

### Adipose-derived stem cell (ADSC) preparation

The ASCs were prepared from another 4 donor rats not involved in the experimental design as the transplanted cells were non-autologous. Adipose tissues from the abdominal wall of rats were obtained and then placed into a labeled sterile tube containing 15 mL of a phosphate-buffered solution (PBS; Gibco/Invitrogen, Grand Island, NY, USA). Enzymatic digestion was performed using 0.075% collagenase II (SERVA Electrophoresis GmbH, Heidelberg, Germany) in Hank’s Balanced Salt Solution for 60 min at 37°C with shaking. Digested tissue was filtered and centrifuged, and erythrocytes were removed by treatment with an erythrocyte lysis buffer. The cells were transferred to tissue culture flasks with Dulbecco’s modified Eagle’s medium (DMEM, Gibco/BRL, Grand Island, New York, USA) supplemented with 10% fetal bovine serum (FBS) (Gibco/BRL) and after an attachment period of 24 h, non-adherent cells were removed by a PBS wash. The attached cells were cultured in DMEM supplemented with 10% FBS, 1% penicillin-streptomycin (Gibco/BRL), and 1.25 mg/L amphotericin B (Gibco/BRL) and expanded in vitro. At 80–90% confluence, cultures were washed twice with PBS and the cells were trypsinized with 0.25% trypsin in 1 mM EDTA (Gibco/BRL) for 5 min at 37°C. After centrifugation, cells were resuspended in a serum-supplemented medium and incubated in a 50 cm^2^ culture flask (Falcon). The resulting cultures were referred to as first-passage cultures and expanded in vitro until passage three. The passage 3 stem cells were washed with PBS and harvested with 0.25% Trypsin/EDTA for 1–2 min at room temperature. The cells were suspended in PBS and centrifuged at 800 rpm for 5 min. This step was repeated. The cells were resuspended in PBS at a density of 1x10^6^/cm^2^. The samples were prepared for immunophenotypic characterization of differentiated ADSCs. Moreover, the third passage cells were confirmed by the 3 linage differentiation (adipogenic, osteogenic, and chondrogenic) [16].

### Labeling stem cells with a green fluorescent protein (GFP)

ADSCs were transfected with non-integrating plasmids containing GFP (addgene, pET His6 GFP TEV LIC cloning vector (1GFP); (Plasmid #29663). One day before transfection, 5 X 10^5^ cultured cells were plated in 1 ml complete growth medium and ADSCs transfected with a single plasmid using the Nucleofector kit (Lonza, Verviers, Belgium) according to the manufacturer’s instructions [17]. ADSCs labeled with GFP were observed using a fluorescence microscope (Leica Microsystems CMS GmbH, Ernst-Leitz-Straße, Wetzlar, D-35578, Germany).

### Immunophenotypic characterization of differentiated ADSCs

ADSCs were initially characterized by their adhesiveness, fusiform morphology, and detection of established surface markers of rat ADSCs by flow cytometry. Following isolation ADSCs were passaged, viable cell counts established, and aliquoted individually at 1 x 10^6^ cells/mL per tube. ADSCs were then incubated with 10 μL of directly conjugated monoclonal antibodies; CD45 PE (rabbit monoclonal; [EP322Y] (ab200315), CD90 PE (mouse monoclonal Antibody (HIS51), bioscience, # 14-0900-81), and CD 105 PE (rabbit polyclonal antibody, CENTER E395; SAB1306487 Sigma Aldrich) at 4°C in the dark for 20 min; matched isotype controls were included for control purposes. Following incubation, 2 mL of PBS containing 2% FCS solution was added to each tube followed by centrifugation for 5 min at 2500 rpm, discarding of the supernatant and resuspending in 500 μL PBS containing 2% FCS. Cell analysis was performed using CYTOMICS FC 500 Flow Cytometer (Beckman Coulter, Brea, CA, USA) and CXP software version 2.2 [18].

### In vitro adipogenic, osteogenic, and chondrogenic differentiation of ADSCs

ADSCs were tested for their ability to undergo tri-lineage differentiation into adipocytes, osteoblasts, and chondrocytes. Passage 4 MSCs at a density of 5 X 10^3^ cells/cm^2^ were seeded into precoated cover glass situated within six-well plates and induced for 3 weeks with either adipogenic (HUXMA-90031; Cyagen Biosciences Inc., Guangzhou, China), osteogenic (#HUXMA-90021; Cyagen Biosciences Inc.), or chondrogenic (#HUXMA-90041; Cyagen Biosciences Inc.) differentiation media, respectively. The cells were then fixed in 10% formalin and stained with either Oil Red O, Alizarin red, or Alcian blue according to standard procedures [19].

### Isolation of platelet-rich plasma (PRP)

PRP donor animals (n = 7) were sacrificed and each was used to treat two rats involved in this study. PRP was prepared using a double-spin technique. Whole blood was drawn from rats by an intra-cardiac puncture. The blood was collected in vacuum tubes containing citrate phosphate dextrose. The whole blood was then centrifuged at 160×g for 10 min, and the supernatant was transmitted to another vacuum tube. The supernatant was exposed to another centrifugation for 15 min at 250×g to produce platelet-poor plasma (PPP) and PRP. The topmost layer, which consisted of the PPP, was aspirated and placed into a new tube. The remaining layer was the PRP. Approximately 400 μL of PRP was activated with 40 μL of 10% CaCl2 to form a gel for application to prevent leakage from the wound [20]. Platelet concentration of PRP was determined using an automated cell counter. Aliquots from each section preparation were examined for platelet cell count to confirm that the concentration was at least 3 times higher than the whole blood values.

### Induction of DM

Type I diabetes was induced in overnight fasted rats by a single intraperitoneal (IP) injection of freshly prepared Streptozotocin (STZ powder was obtained from Sigma-Aldrich Chemical Co., St. Louis, MO, USA; 60 mg/kg, dissolved in 0.1M cold citrate buffer, pH 4.5). After STZ injection, rats acquired drinking water containing sucrose (15 g/L) for 48 h, to lessen the early death due to insulin discharge from partially injured pancreatic islets. Seventy-two hours later, rats were checked for hyperglycemia, and those with fasting blood sugar more than 250 mg/dL were included in the study. Diabetic rats received long-acting insulin (2–4 U/rat) via subcutaneous injection to maintain blood glucose levels in a desirable range (350 mg/dL) and to avoid subsequent development of ketonuria [21]. Animals were maintained in a diabetic state for 6 weeks before the start of the wound-healing experiment.

### Wound model

Galiano’s murine healing model was used [22] as this model minimizes rodent wound contractions and therefore mimics the wound healing processes occurring in humans including granulation tissue formation and reepithelialization. Rats were anesthetized with isoflurane gas (SEDICO, Egypt) inhalation (2.5% in 500 ml/min of air), and surgeries were performed under standard sterile conditions. Two circular, full-thickness 5-mm diameter cutaneous wounds were inflected on the back of each rat, and sterile donut-shaped silicone splints with a diameter two times of the wound were fixed to the surrounding wound edge with an adhesive film (3M™ Steri-Strip™ Skin Closures, 3M Science, Egypt) and interrupted 6-0 silk thread sutures to prevent skin retraction. The wounds were then covered with a semi-occlusive dressing (3M Tegaderm®, Egypt). During all the experiments, rats daily received intraperitoneal injection of buprenorphine (0.1 mg/kg/day).

### Wound closure analysis

Wound closures were blinded quantified through the measure of the wound reepithelialization at day 3, day 7, day 10, and day 14 post-surgery, through a macroscopic analysis of the lesions on the back of rats. A disposable 10-cm medical paper wound measuring ruler was used to measure the wound size. The wound area % at day X postsurgery was calculated as follows: (wound area at day X/Wound area at corresponding day 0) X 100 [9].

### Experimental design and treatment protocol

**Fig. 1:**
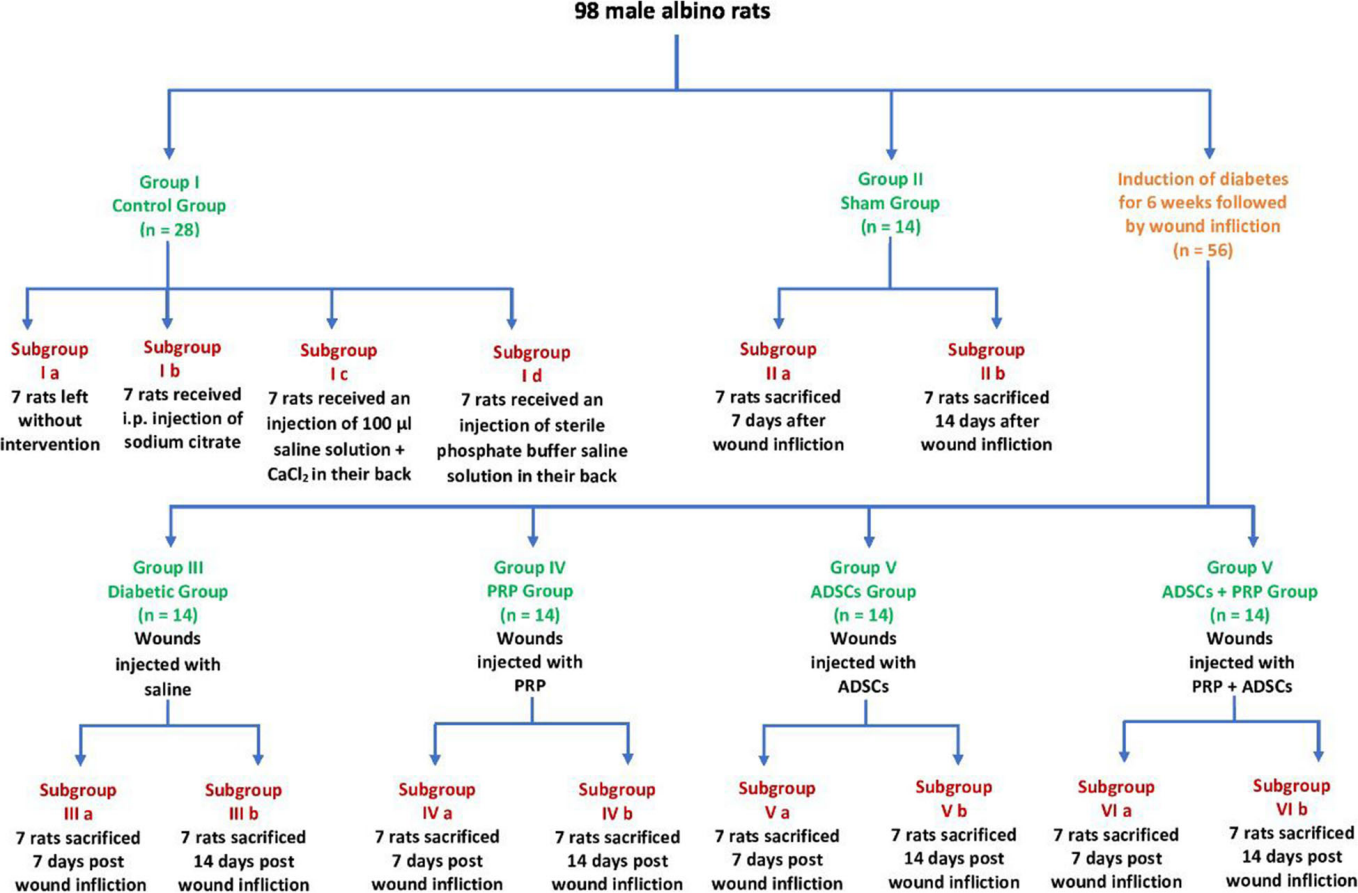
Schematic representation of the experimental design

The experimental design is shown in Fig. 1. Ninety-eight male rats were randomly divided into six groups as follows:

Group I (control group, unwounded group; n = 28): Rats were fed a regular chow diet for 6 weeks. The rats were divided equally into three subgroups of 7 rats each:

Subgroup Ia: The rats were left without any intervention.

Subgroup Ib: The rats were injected intraperitoneal with a single dose of 0.25 mL/kg body weight sodium citrate buffer (vehicle for STZ).

Subgroup Ic: Rats were injected with 100 μl saline solution + CaCl_2_ on the back of each rat.

Subgroup Id: Rats were injected with sterile phosphate-buffered saline solution on the back of each rat.

Group II (Sham operation group, wounded, and non-diabetic; n = 14): Rats were fed a regular chow diet for 6 weeks, then the wound was inflected at the back of each rat, and immediately after injury, the wound base and edges were injected with 100 μl saline.

Group III (diabetic group; n=14): After 6 weeks of DM induction, the wound was inflected on the back of each rat, and immediately after injury, the wound base and edges were injected with 100 μl saline.

Group IV (DM+PRP group; n= 14): After 6 weeks of DM induction, the wound was inflected on the back of each rat, and immediately after that, the wound base and edges were injected with 4 mL PRP activated with 10% CaCl_2_.

Group V (DM+ADSCs group; n= 14): After 6 weeks of DM induction, the wound was inflected on the back of each rat, and immediately after that, the wound base and edges were injected with 100 μl of saline solution containing 2 × 10^6^ ADSCs.

Group VI: (DM+ADSCs +PRP group; n= 14): After 6 weeks of DM induction, the wound was inflected on the back of each rat, and immediately after that, the wound base and edges were injected with 100 μl of saline solution containing 2 × 10^6^ ADSCs—in combination with 4 mL PRP activated with 10% CaCl_2_.

### Sampling

Macroscopic pictures of wounds at different time intervals were captured through the experiment. Rats in each group (except the control group) were equally subdivided into two subgroups (a & b) as follow: Rats in subgroup a were sacrificed after 7 days of wound inflection to assess the inflammatory phase of wound healing while rats in subgroup b were sacrificed after 14 days of wound induction to assess the proliferative phase of wound healing. In each subgroup, the samples were taken from the wound site of the ulcerative tissue.

Half of the skin tissues were collected from rats to be evaluated by light microscopy with hematoxylin and eosin (H&E) and Masson’s trichrome staining. An immunohistochemical evaluation for PCNA and CD31were also performed. The other half of skin fresh tissue specimens were kept frozen at −80°C for later quantitative real-time polymerase chain reaction (qRT-PCR) to assess the gene expression of Notch1, Dll4, Jag1, Hes1, Hey1, VEGF, and SDF-1&EPSCm (ß1 Integrin). Western blot analysis was also performed to assess the protein expression of Notch 1, Jag1, and Hes1.

### Gene expression profile

Total RNA was extracted from the skin specimens of treated and control rats using TRIzol (Invitrogen) according to the manufacturer’s instructions. The concentration and purity of extracted RNA were measured by the Nano-Drop 2000C spectrophotometer (Thermo Scientific, USA). At absorbance ratio A260/A280, RNA purity for all samples was > 1.9. The integrity of RNA was verified on 2% agarose gel using a gel electrophoresis image (Gel Doc. BioRad) [23]. Complementary DNA (cDNA) was synthesized for the target genes using SensiFast cDNA synthesis kits (Sigma Bioline, UK) according to the manufacturer’s instruction using a T100 Thermal Cycler (Bio-Rad, USA).

Quantitative PCR was performed using Maxima SYBR Green/ROX qPCR master mix (2x) (Thermo Scientific, USA) [24]. Primer pairs for selected target and reference genes (Notch 1, Dll4, Hes1, Hey1, Jag1, VEGF, SDF1, EPSCm, and GAPDH) were purchased from Genwez (New Jersey, USA) (Table 1). Each PCR reaction consisted of 500 ng per reaction of cDNA (except for NTC and cDNA control), 12.5 μl Maxima SYBR Green qPCR Master Mix (Maxima SYBR Green qPCR, ThermoFisher Scientific), 0.3 μmol l−1 of each forward and reverse primer, 10 nmol l−1/100 Nm ROX Solution, and nucleases-free water to a final volume of 25 μl. The reaction was completed in AriaMx Real-Time PCR (Agilent Technologies, USA) using a two steps protocol: initial denaturation at 95°C for 10 min, then 40 cycles of denaturation at 95°C for 15 s followed by annealing/extension at 60°C for 60s. A melting curve protocol was run at the end of the PCR by heating at 95°C for 30 s followed by a 65°C for 30 s and 95°C for 30 s. The expression levels of target genes were normalized to the housekeeping gene glyceraldehyde-3-phosphate dehydrogenase (GAPDH). Relative gene expression ratios (RQ) between treated and control groups were calculated using the formula: RQ = 2^-ΔΔCt^ [33].

**Table 1:** Primers used for SYBR green quantitative real-time PCR

Gene	Sequences (5’->3’)	References	Accession
**Notch1**	CCA GCA GAT GAT CTT CCC GTA C ACT GCC GCT ATT CTT GTC CC	[25]	XM_032903023.1
**DII-4**	TTC CAG GCAACC TTC TCC GA ACT GCC GCT ATT CTT GTC CC	[26]	XM_032903968.1
**Jagged-1**	CCT CGG GTC AGT TTG AGC TG CCT TGA GGC ACA CTT TGA AGT A	[27]	XM_032904296.1
**Hes1**	CCA GCC AGT GTC AAC ACG A AAT GCC GGG AGC TAT CTT TCT	[28]	XM_032900059.1
**Hey1**	GCG CGG ACG AGAATG GAAA TCA GGT GAT CCA CAG TCA TCT G	[28]	NM_010423.2
**VEGF**	GTACCTCCACCATGCCAAGT TCACATCTGCAAGTACGTTCG	[29]	XM_032900655.1
**SDF-1**	GAG AGC CAC ATC GCC AGA G TTT CGG GTC AAT GCA CAC TTG	[30]	AF189724.1
**EPSCm**	GACCTGCCTTGGTGTCTGTGC AGCAACCACACCAGCTACAAT	[31]	XM_032888182.1
**GAPDH**	AGT TCA ACG GCA CAG TCA A TAC TCA GCA CCA GCA TCA CC	[32]	XM_032902285.1

### Western blot

Anti-rabbit polyclonal antibodies against Notch 1 (Abcam, ab8925), anti-rabbit monoclonal antibodies against Jag1 (Abcam, ab109536, [EPR4290]), anti-rabbit monoclonal antibody against Hes1 (Abcam, ab95439, [EPR4226]), and anti-rabbit polyclonal antibodies against GAPDH were used. Protein extraction was performed using ice-cold 1X cell lysis buffer containing 50 mmol Tris (SRL, Mumbai, India), 1 mmol EDTA (Fischer Scientific, New York), 150 mmol NaCl (Sigma Aldrich), 1 mol sodium fluoride (Fisher Scientific, Qualigens, Mumbai), 0.1% sodium dodecyl sulfate (SDS; Fisher Scientific), 1% Triton X-100 (Sigma Aldrich), 2 mmol phenylmethylsulfonyl fluoride (Sigma-Aldrich), and 4% protease inhibitor cocktail (Roche Diagnostics, Manheim, Germany). Whole tissue specimens were minced and homogenized by passing through 20G, 22G, and 26G hypodermic needles.

The resultant cell lysates were agitated on ice for 30 min followed by centrifugation at 21,000g for 30 min to collect the supernatant. Protein concentration was estimated by the Folin-Lowry method using a spectrophotometer (Beckman Coulter Inc., Indianapolis, Indiana). The extracted protein was incubated in Laemmli buffer for 10 min at 95°C. Protein (50 mg loading) was resolved using 10% SDS (sodium dodecyl sulfate) polyacrylamide gel electrophoresis and transferred onto polyvinylidene difluoride membrane (Amersham Biosciences, Bucks, UK). The blot was blocked with 5% nonfat dry milk (NFDM) in 1% TBS with Tween 20 overnight at 4°C. The membrane was incubated with anti-Notch 1, Jag1, and Hes1 and GAPDH antibodies (1:500; Ab3209, Millipore) at room temperature for 2 h followed by incubation with goat anti-rabbit horseradish peroxidase-conjugated secondary antibody (1:5000, Millipore) for 2 h at room temperature.

Detection was performed with Super Signal West Femto substrate (Thermo Scientific, Waltham, Massachusetts) on photographic films (Eastman Kodak Co., Rochester, New York). The later blot was stripped using stripping buffer (62.5 mmol Tris, 2% DS, 100 mmol *β*-Mercaptoethanol) for 10 min at 60°C to detect housekeeping protein. GAPDH was used as housekeeping protein and detected using MAB1501 (Millipore) at a 1:5000 dilutions. After washing twice with 1X TBST, densitometric analysis of the immunoblots was performed using Image analysis software on the Chemi Doc MP imaging system (Version 3) produced by Bio-Rad (Hercules, CA).

### Histological analysis

At the end of the experiment, the rats were anesthetized by sodium thiopental (40 mg/kg IP) after 12 h of fasting. Then, vascular perfusion fixation through the left ventricle was performed. The rats were fixed on an operating table to take skin specimens. The half of the skin tissue collected from rats in all groups was fixed in 10% buffered formol saline, embedded in paraffin, and sectioned at 4.0 μm. The sections were dehydrated with successive concentrations of ethanol and washed twice in distilled water. The sections of the skin tissue at days 7 and 14 were stained with hematoxylin and eosin (H&E) and with Masson’s trichrome in accordance with the protocols of the manufacturer to detect the reepithelialization/granulation tissue formation and collagen deposition, respectively. Finally, the histological sections were observed and analyzed under a microscope (Leica DMR 3000; Leica Microsystem) by two blinded experienced investigators [34].

### Immunohistochemical staining

For detection of new vessel formation, the wound areas were analyzed using CD31 primary antibody (rabbit monoclonal primary antibody, SAB5500059, Sigma Aldrich, USA). For the detection of basal keratinocyte proliferation, the PCNA primary antibody was used (rabbit polyclonal Anti-Proliferating Cell Nuclear Antigen antibody, SAB2701819, Sigma-Aldrich, USA). For detection of adipose-derived MSCs (ADSCs) in cutaneous tissues after injection in both treated groups—group IV (DM+ADSCs) and group V (DM+ADSCs+PRP), the CD105 antibody (rabbit, polyclonal primary antibody, SAB1306487, Sigma Aldrich, USA) was used.

Briefly, after fixing, embedding in paraffin, and dewaxing, the tissue sections were blocked in 3% normal goat serum/0.3% Triton X-100/0.1% BSA (Sigma Aldrich) in PBS. The sections were then incubated for 24 h at 4°C with the primary antibody (primary) against CD31 & PCNA, respectively (1:100 dilution), followed by goat anti-mouse IgG (secondary) for each primary antibody for 1 h at room temperature. After hematoxylin staining, tissue sections were washed and then dehydrated with ethanol, treated with dimethylbenzene, and sealed for microscopic analysis by two blinded experienced investigators [34].

### Morphometric study

The mean area percentage of collagen fiber deposition as indicated by Masson’s trichrome staining, the mean area percentage of PCNA, and the number of CD31-positive vessels were quantified to detect the neoangiogenesis, and CD105 surface marker of ADSCs was measured in five images from five non-overlapping fields from each rat of each group using the Image-Pro Plus program version 6.0 (Media Cybernetics Inc., Bethesda, Maryland, USA).

### Statistical analysis

Statistical analysis was performed using the statistical software package SPSS for Windows (Version 16.0; SPSS Inc., Chicago, IL, USA). GraphPad Prism 8.0.2 (GraphPad Software, SanDiego, CA, USA) was used for graphical representation. Differences between groups were evaluated using one-way analysis of variance (ANOVA) followed by Tukey test, and Kruskal-Wallis followed by Mann-Whitney *U* test regarding parametric and non-parametric data, respectively. Data are expressed as mean ± standard error (SEM) (parametric) and median (non-parametric), Spearmen correlations were applied for correlating different variables, and p value <0.05 was considered significant.

## Results

### Confirmation of adipose-derived MSC isolation

**Fig. 2:**
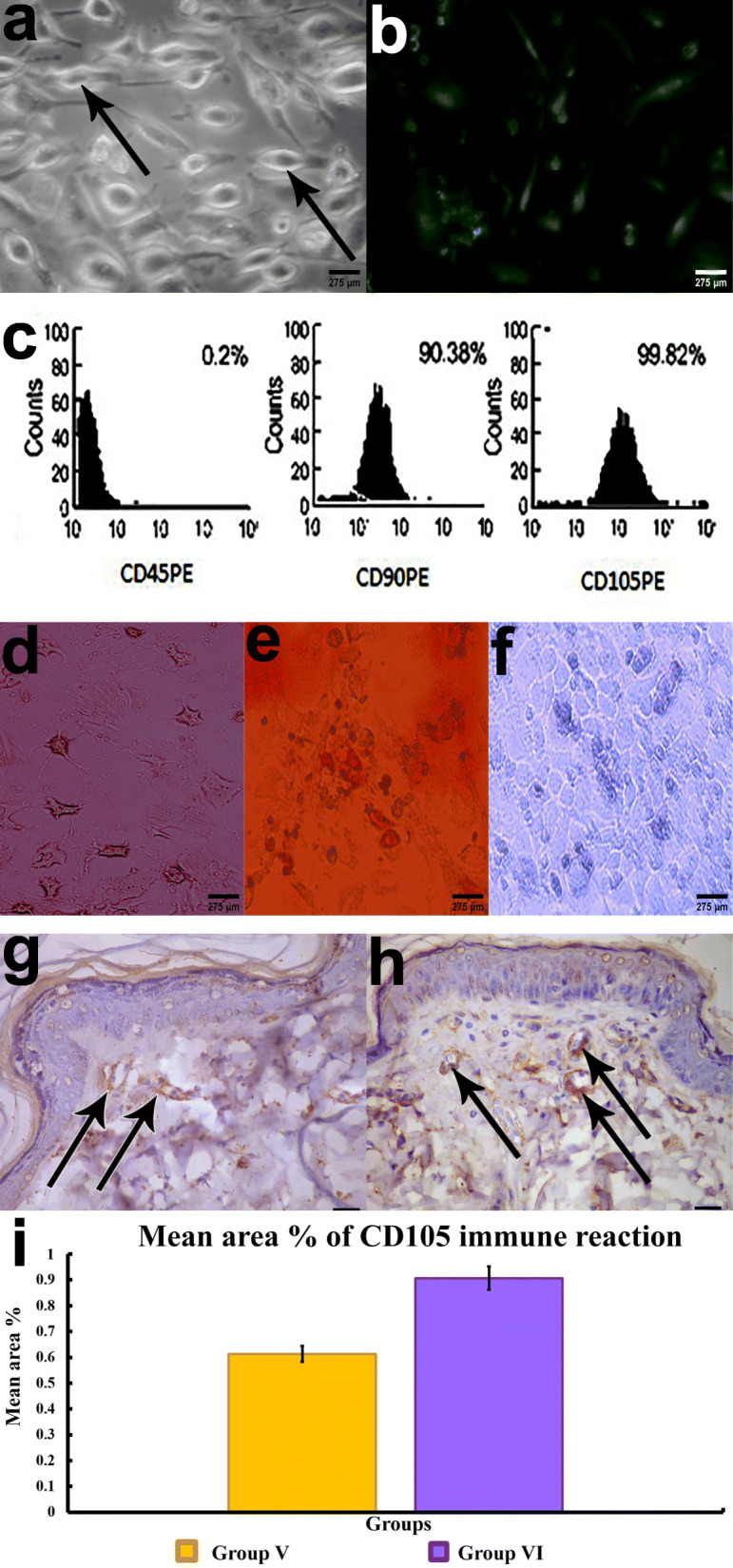
**A** An inverted microscope micrograph from primary culture of mesenchymal stem cells. **B** Fluorescent microscopic image demonstrating fluorescence of MSCs labeled with GFP 2 weeks after implantation. **C** Flow cytometry analysis of surface antigens of MSCs; CD45: 0.4%, CD90: 92.33% and CD105: 97.25%. **D** Osteogenesis differentiation stained with von Kossa stain and its control. **E** Adipogenesis differentiation stained with Oil Red O stain and its control. **F** Chondrogenesis differentiation stained with Alcian blue stain and its control. **G**, **H** Immuno-expression of CD105 in groups IV and V. **I** Mean area percentage of CD105 expression in the different experimental groups

ADSCs were initially identified after 2 weeks’ isolation in culture by an inverted microscope as spindle-shaped cells between rounded cells (Fig. 2A) and injected ADSCs labeled with GFP were observed using a fluorescent microscope (Fig. 2B). Cell surface marker expression confirmed ADSCs identity via CD90 (92.33% positive expression), CD 105 (97.25% positive), and CD45 (0.4%) being consistent with ISCT MSC guidelines [35] (Fig. 2C). Confirmation of adipogenic, osteogenic, and chondrogenic differentiation was also established (Fig. 2D–F). Moreover, CD105 immunoexpression was investigated in cutaneous tissue 2 weeks post-transplantation as it is a type I membrane glycoprotein receptor which increased upon culture passages of MSCs. So, supporting evidence for homing of ADSCs in cutaneous tissue, the immune expression of CD105 (a surface marker of ADSCs) were detected in treated groups; group IV (DM+ADSCs) and group V (DM+ADSCs+PRP), with better immune-expression in group V indicating better homing with combined therapy of PRP with ADSCs (Fig. 2G–I).

### Enhanced wound closure rate in the ADSCS+PRP group via modulating Notch signaling pathway

**Fig. 3:**
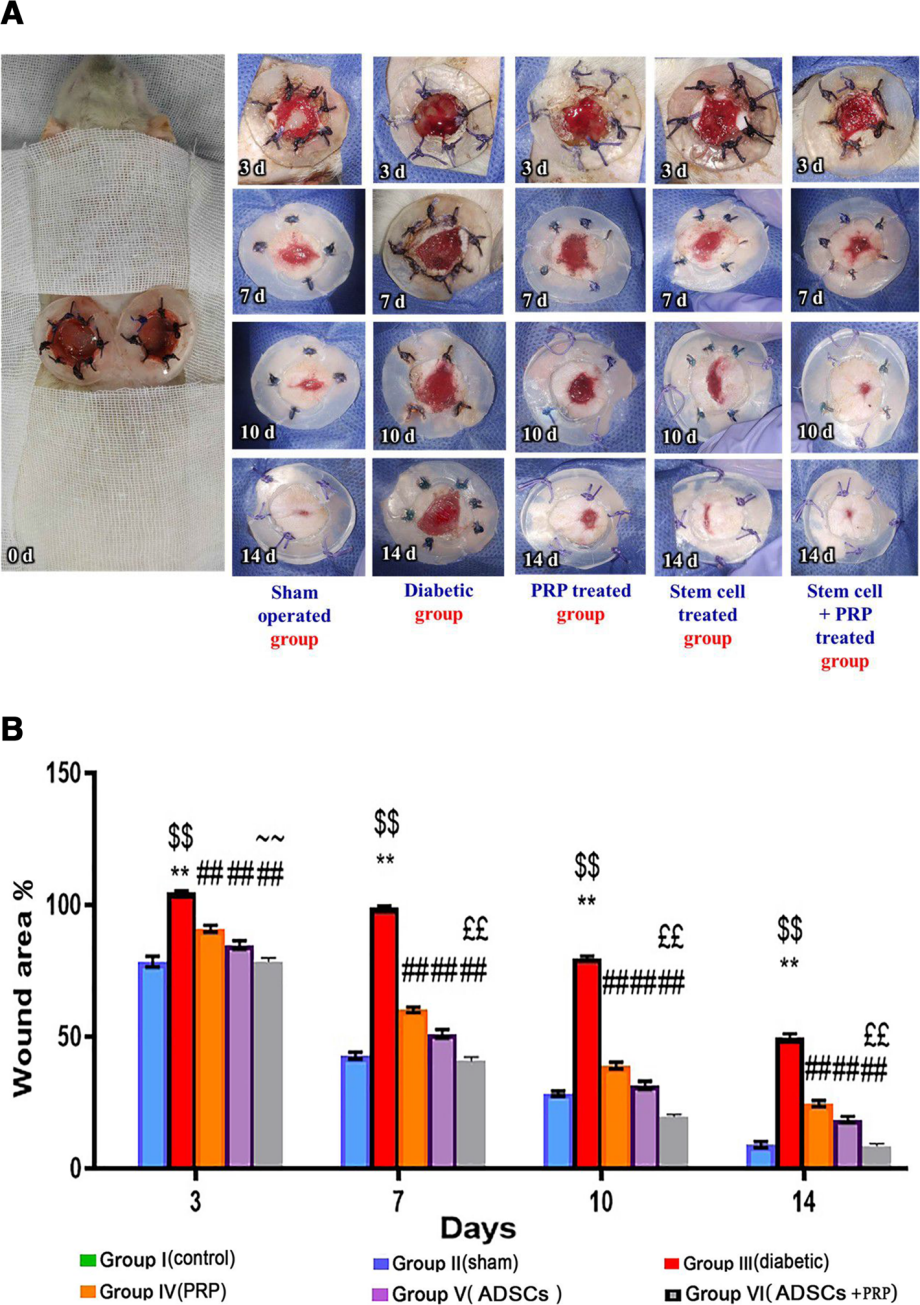
**A** Macroscopic representation of wounds among different experimental groups. Sham group (wounded and non-diabetic group): on the 3rd day of healing appeared as continuous filling of the wound periphery with healthy stable granulation tissue which then rapidly infiltrated all the wound surface and hastily covered by epithelium to decrease the wound surface area, at the end of the study the wound appeared nearly completely healed. Diabetic group: filling with granulation tissue was developed slowly, the formed tissue was unstable easily ulcerated, the epithelium was also poorly established, and the wound failed to be healed by the end of the study with persistent cardinal signs of inflammation. PRP-treated group: although granulation tissue gradually filled the wound, about 15% of the wound surface was still unhealed by the end of the study. Stem cell-treated group: showed continuous filling of the wound surface with healthy granulation tissue that rapidly covered by epithelium to decrease the wound surface area, but about 10% of wound surface was still unhealed by the end of the study. Stem cells + PRP-treated group: healing characteristics and wound development were closely resembling that in the sham group and by the end of the study, the wound appeared nearly completely healed. **B** Photograph represents wound area percentage. n= 7 per group. Data are expressed as mean±SE. **Significant compared to the control group at p<0.01, ^$$^significant compared to the Sham group at p<0.01, ^##^significant compared to the diabetic group at p<0.01, ^££^significant compared to groups IV and V at p<0.01, and ~~significant compared to group IV at p<0.01

As depicted in Fig. 3A, the wound size was markedly reduced in all treatment groups and sham-operated ones compared with the diabetic group. The diabetic wound was easily ulcerated during several attempts of dressing and leaving yellowish-red wounded surface. Shame and group treated with PRP-ADSCs were considered the best-healed groups showing accelerated filling of the wound periphery with healthy rosy red and reddish granulation tissue followed by gradual epithelization and nearly complete healing at the 14th day. The ADSC-treated group was considered the second-best healing group and the PRP-treated group showing less grade of healing but better than the diabetic group.

Cardinal signs of inflammations obviously appeared in the diabetic group at all times of treatment. Cardinal signs of inflammation including painful touching, redness, and swelling of neighboring skin surrounding the wound edges. Sham and group treated with PRP-ADSCs showed the least cardinal signs of inflammation which ceased with the end of the study.

The mean wound area% at days 3, 7, 10, and 14 post wound inflection was 78.4 ± 2.0, 42.77 ± 1.3, 28.55 ± 0.9, and 9.08 ± 1.1, respectively, in the sham group (group II), while in the diabetic group (group III), the wound area% was significantly increased (104.7 ± 0.59, 99.05 ± 0.53, 79.75 ± 0.78, and 49.55 ± 1.4 on days 3, 7, 10, and 14, respectively) compared to the sham group, reflecting lower wound closure rates compared to the sham group. In the treated groups, the wound area was 90.98 ± 1.28, 60.18 ± 0.98, 39.03 ± 1.2, and 24.68 ± 1.16% in the PRP group (group IV), 84.93 ± 1.6, 51.13 ± 1.6, 31.63 ± 1.5, and 18.6 ± 1.15 % in the ADSCs group (group V), and 78.55 ± 1.3, 44.77 ± 1.4, 19.83 ± 0.8, and 8.5 ± 0.9 in the PRP+ADSC group (group VI) at days 3, 7, 10, and 14 days, respectively (Fig. 3B).

### Histological findings underlying the accelerated diabetic wound healing treated with ADSCs combined with PRP via modulating Notch signaling pathway

#### H&E results

**Fig. 4:**
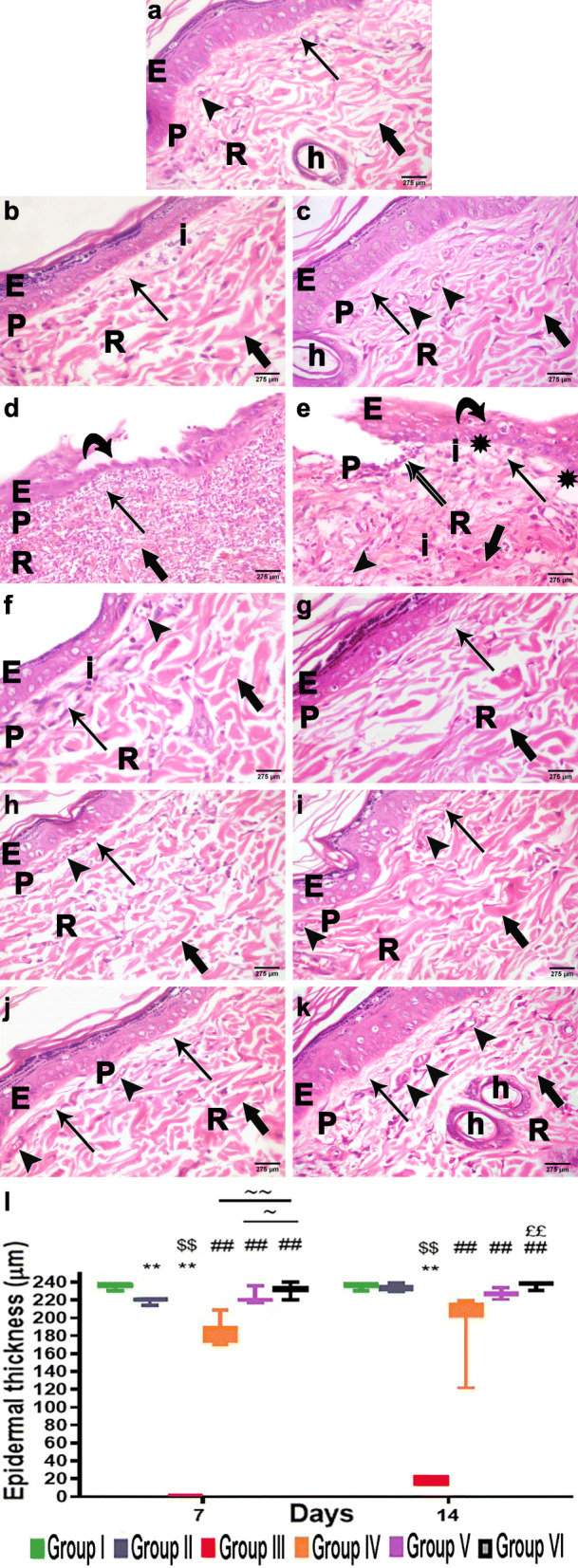
The H&E-stained skin sections revealed that PRP+ADSC-treatment accelerate the diabetic wound healing: **A** group I (unwounded group) showed the normal epidermis (E) and dermis with its papillary (P) and reticular (R) layers. **B** Group II (wounded and non-diabetic group); IIa showed the thin epidermis (E) with inflammatory cell infiltration (I) in papillary layer (P). **C** Group IIb showed near-normal epidermis (E) and dermis with its papillary (P) and reticular layers (R). **D**, **E** Group III (diabetic group) showed the interrupted epidermis with and disorganized keratinocytes with vacuolated cytoplasm (V) and absence of keratin, wound beds were covered by a single layer of squamous cells (curved arrow). The dermis showed a massive inflammatory infiltration, and minimal fine collagen deposition (arrow) with areas of deficient collagen deposition (asterisk). The reticular (R) layer has thick collagen bundles (bold arrow). **F**–**K** All treated groups (DM+ PRP group, DM+ADSC group, and DM+ADSCs +PRP group): showed intact epidermis (E) with keratohyalin granules and keratin with papillary (P) layer showed fine collagen fibers (arrow), and blood capillaries (arrowhead). The reticular (R) layer has thick collagen bundles (bold arrow). **l** Epidermal thickness of the different experimental groups. n = 7 per group. Data are expressed as median (maximum and minimum), **significant compared to the control group at p<0.01, ^$$^significant compared to the Sham group at p<0.01, ^##^significant compared to the diabetic group at p<0.01, ^££^significant compared to groups IV and V at p<0.01, ~significant compared to group V at p<0.05, and ~~significant compared to group IV at p<0.01

Examination of H&E stained sections of the control unwounded group (group I) demonstrated the normal histological layers and structure of thin skin. The epidermis was composed of a keratinized stratified squamous epithelium, consisting predominantly of keratinocytes arranged in four strata. Immediately beneath the epidermis, the dermis was seen formed of two layers. The superficial papillary layer contained thin interlacing collagen fibers, connective tissue cells, and numerous blood capillaries. The deeper reticular layer was thicker than the papillary layer, and poorly demarcated from it. It was less cellular and formed of thicker collagen bundles running in different directions (Fig. 4A).

Examination of H&E stained sections taken 7 days post wound infliction in the sham group, wounded and non-diabetic (group IIa) revealed healed wounds with an intact epidermis and dermis. The epidermis appeared thinner than that of the control group and was arranged in three layers. The papillary layer of the dermis was thin with minimal collagen deposition and inflammatory cell infiltration. Collagen bundles in the reticular layer were thicker than that of the control group arranged parallel to the surface (Fig. 4B). Sections examined 14 days post wound infliction in the sham group (group IIb) showed similar epidermal thickness and structure to that of the control group. The papillary layer of the dermis showed abundant fine interlacing collagen fibers, fibroblasts with spindle-shaped nuclei, and numerous blood capillaries, with a notable absence of inflammatory infiltration. The reticular layer revealed collagen bundles thicker than those of the control group (Fig. 4C).

The diabetic group (group III) showed delayed wound healing and prominent histological alterations. Sections of the 7th day (group IIIa) showed interrupted epidermis with wound beds covered by a single layer of squamous cells. The epidermis on either side of the wound beds appeared thin with disorganized keratinocytes and notable absence of keratin. Both layers of the dermis showed massive inflammatory infiltration with minimal collagen deposition (Fig. 4D). On the 14th day (group IIIb), the thin diabetic skin revealed persistent interruption of the epidermis. The wound bed contained pyknotic nuclei and the epidermis on either side of the wound appeared disorganized with pyknotic nuclei and a persistent absence of keratin. The papillary layer showed areas of deficient collagen deposition and absence of blood capillaries, while the reticular layer showed thick collagen bundles, and less dense collagen fibers. Both layers showed persistent inflammatory infiltration (Fig. 4E).

Treatment of wounds with PRP (group IV) and ADSCs (group V) both individually and in combination (group VI) leads to acceleration of wound healing when compared to the sham and DM groups at both the 7th and 14th days post wound infliction.

Examination of the PRP-treated group on the 7th day (group IVa) showed healed wounds with intact dermis and epidermis. The epidermis appeared thin with few keratohyalin granules and an absence of keratin. The papillary layer showed areas of fine collagen fiber deposition with inflammatory infiltration and few blood capillaries. The reticular layer showed thick collagen bundles (Fig. 4F). On the 14th day (group IVb), the epidermis appeared thicker with prominent keratohyalin granules and keratin. Collagen of the dermis was arranged in thick bundles parallel to the surface (Fig. 4G). Sections from the ADSC-treated group on the 7th day (group Va) showed intact thin skin. The epidermis appeared thin with few keratohyalin granules and keratin. The papillary layer showed interlacing collagen fibers, blood capillaries, and absence of inflammatory cells, while the reticular layer showed thin collagen bundles running parallel to the surface (Fig. 4H). On the 14th day (group Vb), the epidermis appeared thicker, with more prominent keratohyalin granules and keratin. The papillary layer showed more collagen fibers and blood capillaries, while the reticular layer had thick collagen bundles (Fig. 4I).

The PRP+ADSC-treated group on the 7th day (group VIa) revealed intact thin skin. The epidermis showed all keratinocyte layers. Fine collagen fibers and blood capillaries in the papillary layer, and thick collagen bundles in the reticular layer (Fig. 4J). On the 14th day (group VIb), the epidermis appeared thicker. Abundant, fine, interlacing collagen fibers and numerous blood capillaries were seen in the papillary layer and reticular layers showed thick collagen bundles and numerous hair follicles (Fig. 4K).

#### Masson’s trichrome results

**Fig. 5:**
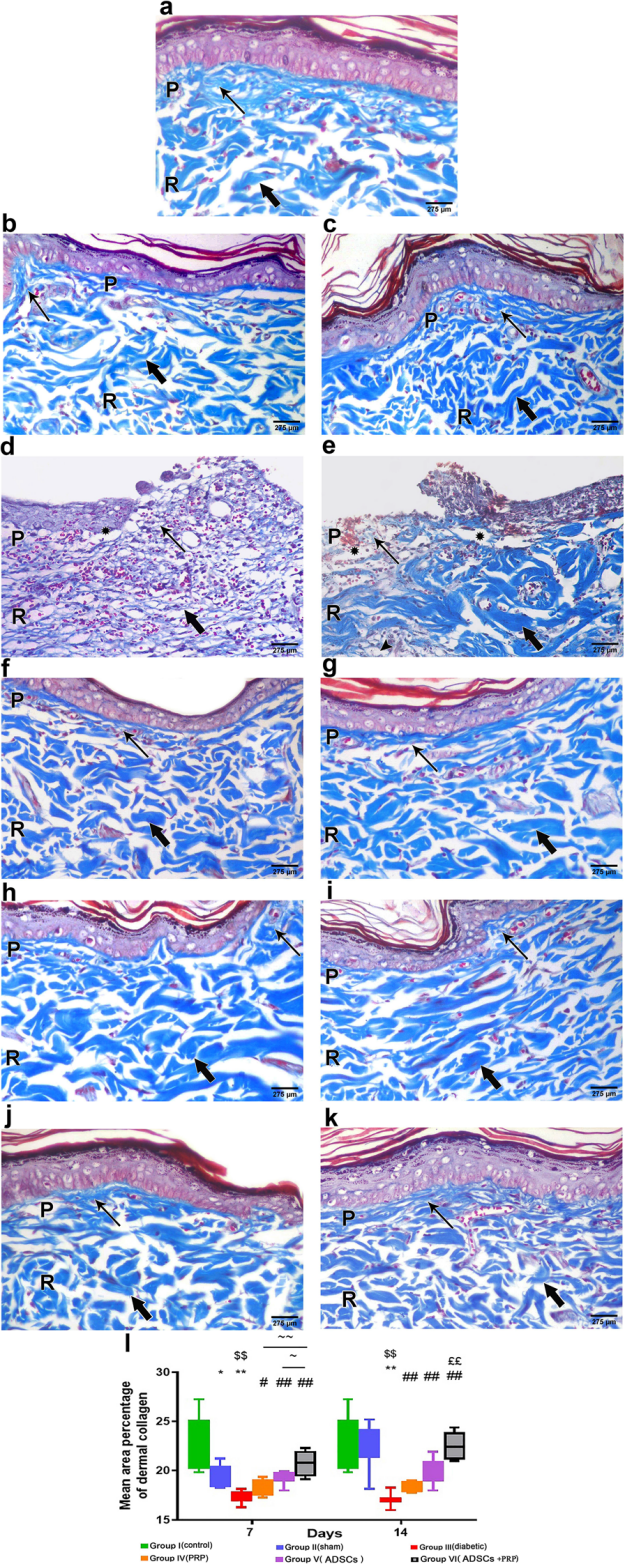
Masson’s trichrome staining of the cutaneous tissue was performed to assess dermal collagen different experimental groups (**A–K**), as there was fine interlacing fibers (arrow) in the papillary (P) layer, and thick, irregular blue bundles (bold arrow parallel) to the surface in the reticular (R) layer. In group III (diabetic group), there were areas of deficient collagen deposition are seen beneath the epidermis (asterisk). **l** Mean area percentage of dermal collagen. n = 7 per group. Data are expressed as median (maximum and minimum), **significant compared to the control group at p<0.01, *significant compared to the control group at p<0.05, ^$$^significant compared to the Sham group at p<0.01, ^#^significant compared to the diabetic group at p<0.05, ^##^significant compared to the diabetic group at p<0.01, ^££^significant compared to groups IV and V at p<0.01, ~significant compared to group V at p<0.05, and ~~significant compared to group IV at p<0.01

In the control unwounded group (group I), dermal collagen was seen as fine interlacing fibers in the papillary layer, and thick, irregular blue bundles in the reticular layer (Fig. 5A). The sham group (wounded and non-diabetic group) on the 7th day (group IIa) showed fine collagen fibers in the papillary layer and thick collagen bundles parallel to the surface in the reticular layer (Fig. 5B). On the 14th day (group IIb), abundant fine interlacing collagen fibers were seen in the papillary layer and thick collagen bundles in the reticular layer (Fig. 5C). The diabetic group (group III) revealed an evident decrease in collagen in both the papillary and reticular layers. On the 7th and 14th day (group IIIa and IIIb), both layers showed pale fine collagen fibers with some areas of deficient collagen deposition. Few areas showed thick collagen bundles on the 14th day (Fig. 5D, E). On the other hand, the treated groups (groups IV, V, and VI; DM+ PRP group DM+ADSC group; and DM+ADSCs +PRP group, respectively) showed a progressive increase in fine collagen fiber deposition in the papillary layer with improvement in the organization of the thick collagen bundles which ran parallel to the surface in the reticular layer. The best collagen deposition and organization was observed in group VI compared to the control group (Fig. 5F–K).

#### Immunohistochemistry staining results

**Fig. 6:**
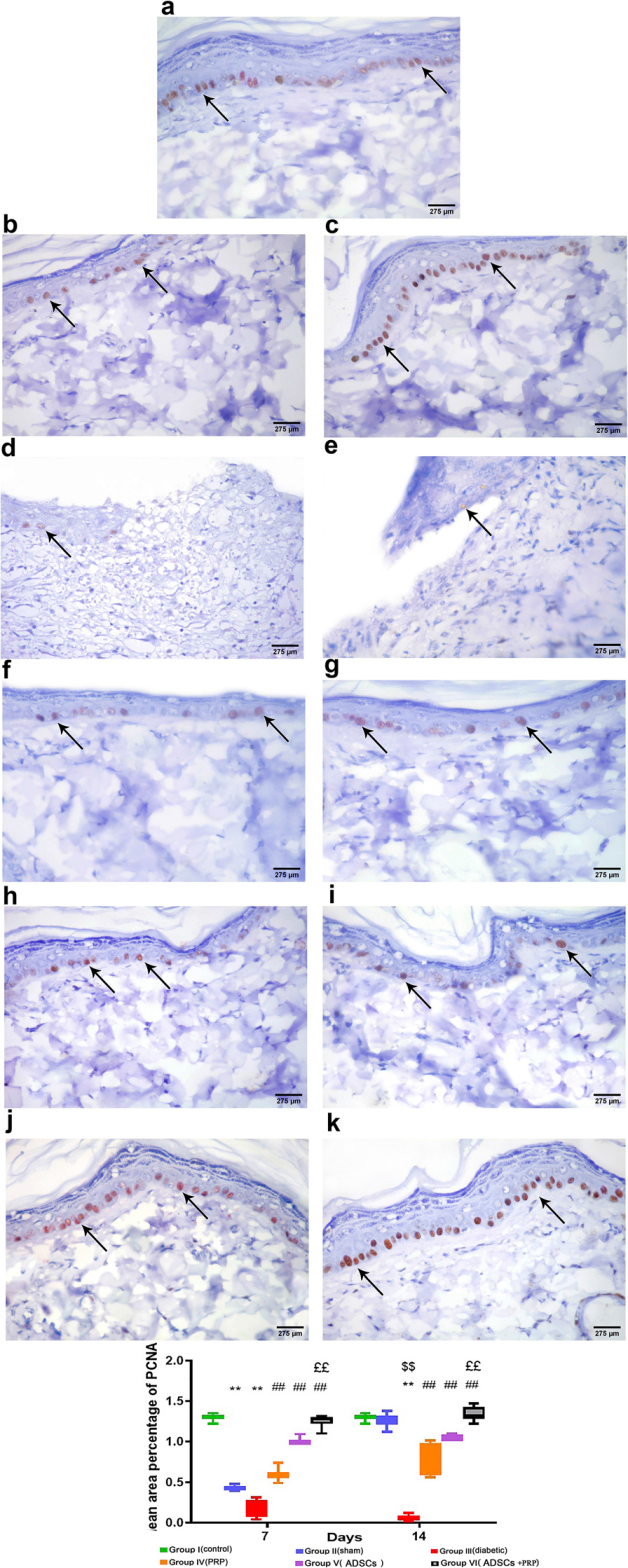
Representative photomicrographs of PCNA immune stained sections showing the basal keratinocytes of the different experimental groups (**A–K**). All groups showed a moderate reaction except group IIb (wounded and non-diabetic group) and VI (DM+ADSCs +PRP group) and showed the intense reaction of PCNA (**C**, **J**, and **K**). **l** Mean area percentage of PCNA expression in the different experimental groups. n= 7 per group. Data are expressed as median (maximum and minimum), **significant compared to the control group at p<0.01, ^$$^significant compared to the Sham group at p<0.01, ^##^significant compared to the diabetic group at p<0.01, ££ significant compared to groups IV and V at p<0.01

Immunohistochemical staining with anti-PCNA antibody was performed to assess cellular proliferation of the epidermis in the wound area. In group I, the basal layer of keratinocytes showed an intense reaction in many cells (Fig. 6A), while in group II, there was a moderate reaction on the 7th day (Fig. 6B) and the reaction became intense on the 14th day in the basal layer of keratinocytes, comparable to that of the control group (Fig. 6C). On the other hand, in group III, a weak reaction was observed in the basal layer of keratinocytes at the 7th and 14th day (Fig. 6D, E). In the treated groups (groups IV, V, and VI), the intensity of the reaction in the basal layer of keratinocytes increased from moderate to intense reaching a reaction comparable to the control on the 14th day of group VI (Fig. 6F–K).

**Fig. 7:**
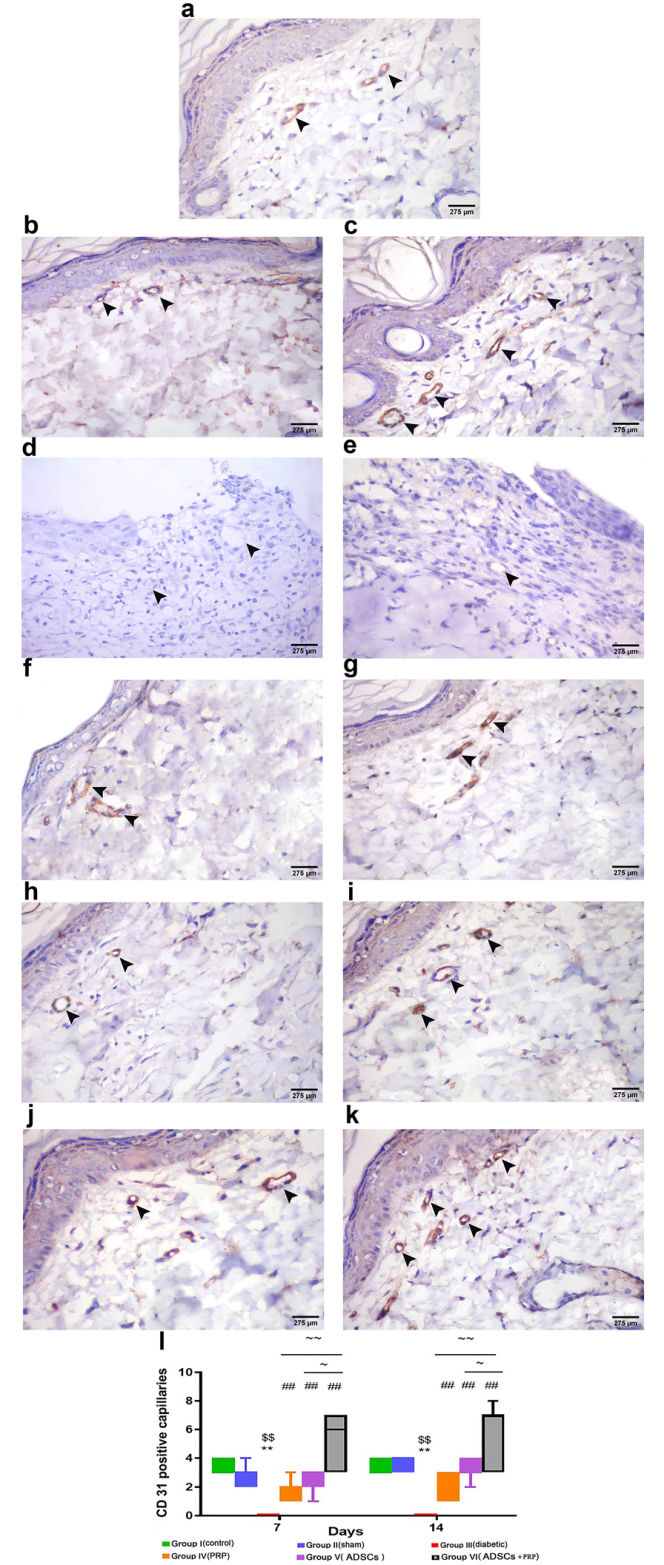
Representative photomicrographs of CD31 immune stained sections showing the endothelial cells of the blood capillaries of different experimental groups (**A**–**K**). **A** Group I (unwounded group): showing a moderate reaction (arrowhead) in the capillaries. **B** Group II (wounded and non-diabetic); **IIa:** showing a weak reaction (arrowhead) in few capillaries. **C** Group IIb: strong reaction (arrowhead) in many capillaries.**D**, **E** Groups III a & b (diabetic groups): negative reaction (arrowhead) in the capillaries. **F** Group IV (DM+ PRP group); IVa: showing a moderate reaction (arrowhead) in some capillaries. **G** Group IVb: showing a strong reaction in the capillaries. **H** Group V (DM+ADSC group); Va: showing a mild reaction in the capillaries. **I** Group Vb: showing a moderate reaction in the capillaries. **J**, **K** Groups VI a & b (DM+ADSCs +PRP group): showing a strong reaction in both days with an increase in the number of capillaries seen on the 14th day. **l** Vascular area density/vascular index in the different experimental groups. n= 7 per group. Data are expressed as median (maximum and minimum), **significant compared to the control group at p<0.01, ^$$^significant compared to the Sham group at p<0.01, ^##^significant compared to the diabetic group at p<0.01, ~significant compared to group V at p<0.05, and ~~significant compared to group IV at p<0.01

Immunohistochemical detection of angiogenesis (new capillary formation) was performed using an endothelial CD31 marker. In the control unwounded group (group I), a moderate CD31 expression was observed in the capillaries of the papillary layer of the dermis (Fig. 7A). In the sham (wounded and non-diabetic) group (group II), CD31 expression was observed on the 7th day which peaked on the 14th day (Fig. 7B, C). In the diabetic group (group III), a negative CD31 reaction was observed on both days (Fig. 7D, E). On the other hand, the treated groups (groups IV, V, and VI) showed new vessel formation with CD31 immuno-expression on the 7th day and peaked on the 14th day (Fig. 6F–K).

#### Morphometric study

In H&E stained sections, the mean epithelial thickness was 236.4±1.1 μm in the control unwounded group (group I), while in the sham (wounded and non-diabetic) group (group II), it was 219.8±1.0 μm on the 7th day and 233.7±1.4 μm on the 14th day. In the diabetic group (group III), the significant decreased epithelial thickness was observed at both days (0.0 ± 0.0 μm and 18.4 ± 1.9 μm on days 7 and 14) respectively when compared to the sham group, the (p<0.05). Furthermore, a significant increased epithelial thickness was observed in all treated groups (groups IV, V, and VI) with the most significant increase observed in group VI when compared to the diabetic group. The mean epithelial thickness of the treated groups was (184.7 ± 5.1, 222.1 ± 2.4, and 231.4 ± 2.4 μm, respectively) on the 7th day and (197.1 ± 12.8, 227.4 ± 1.7, and 237.6 ± 1.2 μm, respectively) on the 14th day (p<0.05) (Fig. 4l).

In Masson trichrome stained sections, the mean area percentage of dermal collagen was (23.2 ± 1.0%) in the control group (group I). In the sham group (group II), it was (19.6 ± 0.4%) on the 7th day and (22.3 ± 0.9%) on the 14th day. In the diabetic group (group III), a significantly decreased collagen deposition was observed (17.4 ± 0.2%) on the 7th (17.1 ± 0.3%) and 14th days when compared to the sham group (p<0.05). Following treatment with PRP and ADSCs, there was a significantly increased collagen deposition in treated groups (IV, V, & VI) with the most significant increase observed in group VI. The mean area percent of collagen deposition of the treated groups on the 7th day was 18.4 ± 0.3, 19.2 ± 0.2, and 20.8 ± 0.5%, respectively (p<0.05), while the mean area percent of collagen deposition on the 14th day were 18.5 ± 0.2, 19.8 ± 0.5, and 22.5 ± 0.5, respectively (p <0.05) (Fig. 5l).

In PCNA stained sections, the mean area percentage of PCNA was 1.3 ± 0.0% in the control group (group I). In the sham group (group II), it was 0.4 ± 0.0% on the 7th day and 1.3 ± 0.0% on the 14th day. In the diabetic group (group III), the significant deceased mean area percentage of PCNA was observed (0.1 ± 0.0%) on both the 7th day and 14th days when compared to group II with (p<0.05). Following the treatment with PRP and ADSCs, there was a significantly increased mean area percentage of PCNA in the treated groups (IV, V, and VI) with the most significant increase observed in group VI. The mean area percent of PCNA on the 7th day were 0.6 ± 0.0, 1.0 ± 0.0, and 1.3 ± 0.0%, respectively (p<0.05), while the mean area percent of PCNA on the 14th day were 0.8 ± 0.1, 1.0 ± 0.0, and 1.3 ± 0.0, respectively (p<0.05) (Fig. 6l).

Morphometric analysis of CD 31 expression showed a CD 31-positive capillary count of (3.4 ± 0.2) in the control group (group I). The granulation tissue of the wound of the sham group (group II) had a count of (2.7 ± 0.3) on the 7th day and (3.6 ± 0.2) on the 14th day. In the diabetic group, there was a significant decrease in capillary count on both days (0.0 ± 0.0) when compared with the sham group (p<0.05). Following treatment with PRP and ADSCs, there was a significant increase in CD 31-positive capillary count in the treated groups (IV, V, and VI) with the most significant increase observed in group VI. The capillary count on the 7th day was 1.9 ± 0.3, 2.4 ± 0.3, and 5.0 ± 0.7, respectively (p<0.05), while at the 14th day was 2.0 ± 0.3, 3.3 ± 0.3, and 5.9 ± 0.8, respectively (p<0.05) (Fig. 7l).

### Effect of PRP and/or ADMSCs on gene expression of Notch1 pathway and angiogenic key elements in rat diabetic wound

To elucidate the role of the Notch signaling pathway relevant to diabetic wound healing, the expression of Notch receptor (Notch1), two Notch ligands (Dll4 & Jag1), and two Notch target genes (Hes1 & Hey1) were detected. qPCR was used to assess the expression of Notch1 pathway-related genes in the diabetic wound.

**Fig. 8:**
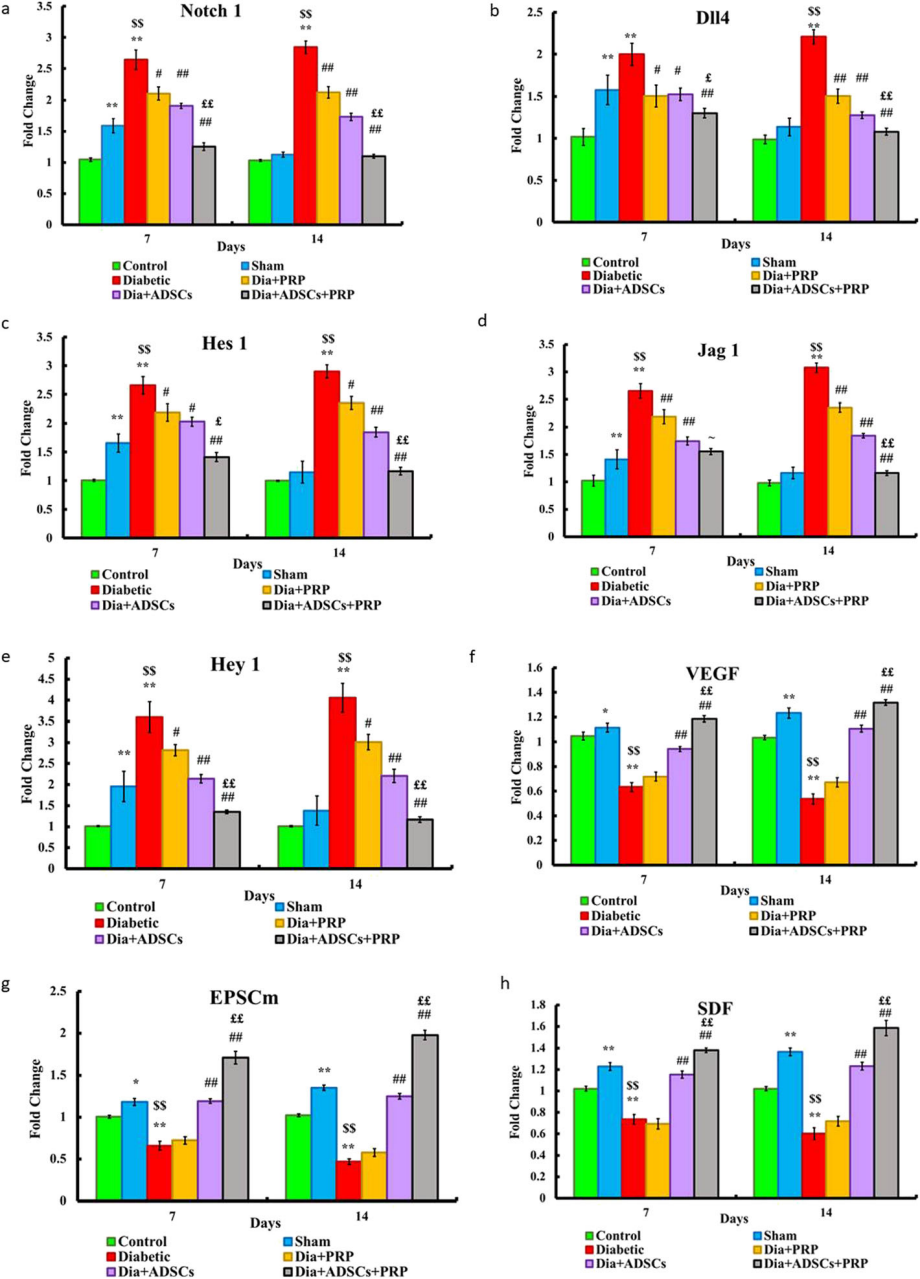
Effect of PRP and/or ADSCs on gene expression of **A** Notch1, **B** Dll4, **C** Hes 1, **D** Jag 1, **E** Hey 1, **F** VEGF, **G** EPSCm, and **H** SDF-1 normalized to GAPDH and expressed as fold of the control. n = 7 per group. Data are expressed as mean±SE, **significant compared to the control group at p<0.01, *significant compared to the control group at p<0.05, ^$$^significant compared to the Sham group at p<0.01, ^#^significant compared to the diabetic group at p<0.05, ^##^significant compared to the diabetic group at p<0.01, ^££^significant compared to groups IV and V at p<0.01, and ~significant compared to group IV at p<0.05

As shown in Fig. 8, the Sham group (group II) at day 7 showed a significant upregulation of Notch1 and its downstream genes including Notch1, Dll4, Jag1, Hes1, and Hey1 when compared to the control group (group I) (p < 0.01). Nevertheless, group II at day 14 showed non-significant upregulation of Notch1 and its downstream genes when compared to group I. In contrast, Notch1 and its downstream genes showed significantly higher expression levels in the diabetic group (group III) than those in group I (p < 0.01). The expression of Notch1 and its downstream genes, in all treated groups (IV, V, and VI), was found to be significantly decreased compared to group III. Group VI at day 14 showed no significant changes in the expression of Notch1 pathway-related genes when compared to either group I or group II. On the other hand, group VI showed significant changes compared to group IV and group V both on day 7 and day 14 (p < 0.01).

Angiogenic gene (VEGF) and epidermal stem cell-related genes (EPSCm and SDF-1) were significantly upregulated in the Sham group (group II) compared with those in the control group (group I) (p < 0.05 for VEGF and SDF-1 and p < 0.01 for EPSCm at day 7, p < 0.05 for the three genes at day 14). The diabetic group (group III) displayed significant downregulation of these genes compared to either group I or II (p < 0.01). Nevertheless, these genes were unchanged between group III and group IV. Further, both groups V and VI displayed significant upregulation of these genes when compared to group III (p<0.01). Moreover, group VI showed significant changes compared to group IV and group V both on day 7 and day 14.

To investigate the relation between Notch1 and the soluble factors (VEGF and SDF-1), Spearman correlation analyses were applied. Notch1 expression levels showed significant inverse correlations with both VEGF (rho=−0.78 and p=0.001) and SDF-1 (rho=−0.72 and p=0.001).

### Effect of PRP and/or ADMSCs on protein levels of Notch1 pathway key elements in rat diabetic wound

Western blot results of Notch1, Jag1, and Hes1 showed a similar trend to that of the gene expression analysis and confirmed it.

**Fig. 9:**
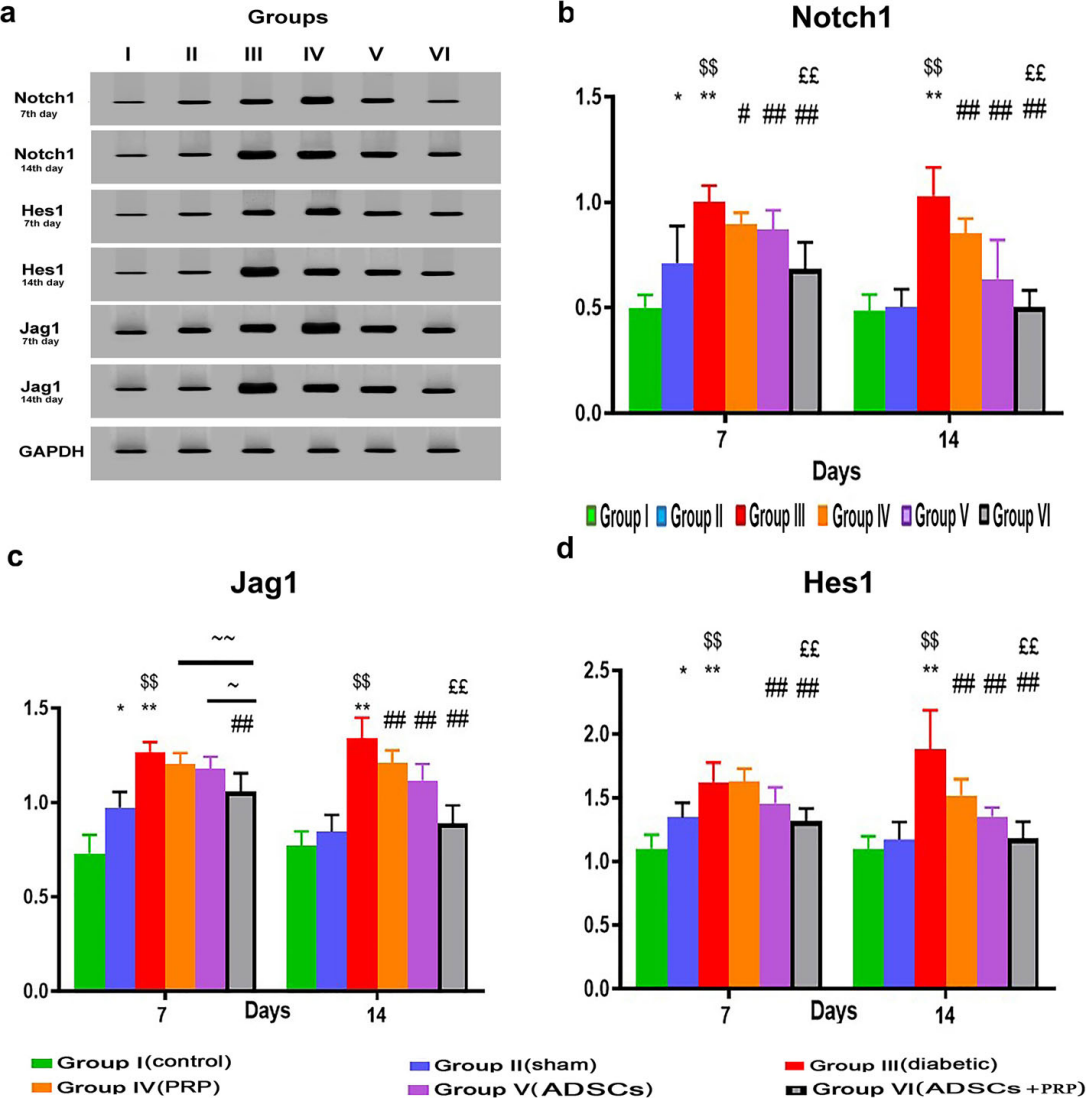
**A** Western blot analysis for measuring Notch1, Hes 1, and Jag 1. GAPDH was used for normalization. **B**–**D** The intensity of immunoreactivity for selected proteins was quantified by densitometry. n= 7 per group. Data are expressed as mean±SE. *Significant compared to the control group at p<0.05, **significant compared to the control group at p<0.01, ^$$^significant compared to the Sham group at p<0.01, ^#^significant compared to the diabetic group at p<0.05, ^##^significant compared to the diabetic group at p<0.01, ^££^significant compared to groups IV and V at p<0.01, and ~significant compared to group IV at p<0.05

As shown in Fig. 9, the Sham group (group II) at day 7 showed a significant upregulation of protein levels of Notch1 (p < 0.05), Hes1 (p < 0.01), and Jag 1 (p < 0.05) when compared to group I. However, on day 14, group II showed a non-significant upregulation of the aforementioned proteins when compared to the control group (group I).

Further, Notch1, Hes1, and Jag 1 showed significantly higher expression levels in group III than those in group I (p < 0.05). The expression of Notch1 in all treated groups (IV, V, and VI) was found to be significantly decreased compared to group III (p < 0.05). The expression of Hes 1 and Jag1 showed a non-significant decrease in groups IV and V and a significant decrease in group VI at day 7, and a significant decrease in all treated groups at day 14 when compared to group III. Group VI at day 14 showed no significant changes in the expression of Notch1 pathway-related genes when compared to either group I or group II (p < 0.05).

## Discussion

Wound healing is definitely a complex process coordinated by numerous molecular events leading to the closure of the wound with or without scar formation. Typically, the events that occur soon after a skin injury could be allocated into four overlapping phases: coagulation and hemostasis, inflammation, proliferation, and remodeling. The proper and coordinated progress of such processes is essential to a normal and effective wound healing. Insufficient wound healing takes place when one or more underlying molecular processes within the different phases are usually disrupted [36]. Therefore, wound healing is a natural response, but in severe or chronic conditions, such as burns and diabetes, this process is insufficient to achieve the effective repair.

Epidermal stem cells (EPSCs) are a multipotent cell type and are committed to the formation and differentiation of the functional epidermis [37]. The microenvironment of stem cells, called “stem cell niches,” performs a key role in regulating the stem cell proliferation, migration and differentiation throughout a network system of numerous interconnected signaling pathways. Among these pathways, the Notch signaling pathway is an essential constituent of stem cell “niches” which plays a vital role in skin development and wound repair. After skin injury, cytokine concentration as well as the extracellular matrix (ECM) components is changed resulting in stem cell niche affection and Notch signaling pathway activation. Hence, the proliferation and differentiation of wound EPSCs are prompted, ultimately contributing to wound healing or scar formation [38].

The sham-operated group of the current study proved that the activation of Notch1 pathway resulted in improved wound closure as evidenced by improved epidermis layer thickness, rejuvenated skin appendages along with more organized and regular collagen fiber arrangement leading to intact epidermis and dermis, which were more or less normal in structure. Moreover, the EPSC marker (ß Integrin), Notch1, and its ligands DLL4 and Jag1, with its downstream target genes Hes1 and Hey1, were significantly increased. Hes1 and Hey1 are considered as chief target genes in the Notch1 signaling pathway, and they play a vital role in maintaining the proliferation potential of EPSCs confirming that the Notch1 signaling plays a significant role in retaining the homeostasis of epidermal epithelial cells. This was confirmed by the significantly increased immune-expression of PCNA in the basal layer of the epidermis compared to the normal skin, which indicated an increased EPSC proliferation. Concomitant with these results, Yang et al. 2016 [39] reported that initial activation of Notch1 signaling throughout wound healing could support EPSC proliferation and preserve their low differentiation potential and even multi-directional differentiation potential. Furthermore, Yang et al. 2016 [39] and Zhou et al. 2018 [40] stated that Notch1 is extensively expressed on EPSCs which were traced mainly in the basal layer of the epidermis, so, if the Notch signaling pathway was blocked, EPSC proliferation would be inhibited hindering the epithelialization process leading to loss of skin barrier function.

Moreover, the sham-operated group showed a significant increased Jag 1 expression, which is the first ligand of the Notch receptor expressed in all skin layers. It plays a significant role in controlling the differentiation of EPSCs [41]. Concomitant with these results, Chigurupati 2007 [42] verified that mice treated with the Notch ligand, Jagged, showed accelerated wound closure (as assessed by surface wound size) suggesting that these effects were mediated by the Notch pathway, so, Jag1 mediated the “dialogue” of Notch signaling in cutaneous tissue, controlling EPSC proliferation, and differentiation as well as playing a role in wound healing and scar formation. This explains that the Notch signaling pathway could affect the biological microenvironment (“niche”) of EPSCs. This could be attributed to the role of the Notch signaling pathway in the angiogenic process. Indeed, vascular endothelial cells express receptors Notch1 and 4 and ligands Delta-like 1 and 4 in addition to Jag1. Thus, Notch1 and Dll4 play a vital role in angiogenic budding. The budding is directed by endothelial tip cells which express high amounts of Dll4. For this purpose, Dll4 is placed at the protruding front directed towards angiogenic signals [43]. Such outcomes were supported by the results of the current study, as evidenced by the improved vascularity in the wound healing process of the sham-operated group, which is confirmed by the significant increase in CD31 immuno-expression indicating new blood vessels formation and by the significant increase in VEGF and STDF-1 gene expression. These results were explained by Chigurupati et al. 2007 [42], who reported that Notch signaling affected several behaviors of vascular endothelial cells which are critical for angiogenesis. Angiogenesis includes endothelial cell migration into the surrounding tissue, cell proliferation, alignment and tube formation, recruitment of parenchymal cells, and a return to quiescence. So, activation of Notch in the wound healing process enhanced vascular endothelial cell proliferation, migration, and tube formation [44].

Regarding the inflammatory phase of the wound healing, the inflammatory cell infiltration of the sham-operated group in the current study was increased during the first week of wound healing confirmed by histological study then decreased after that in the proliferation phase of the wound healing. Consistent with these findings, Kimbal et al. 2017 [45] stated that Notch is critical for the early inflammatory phase of wound healing and directs the production of macrophage-dependent inflammatory mediators. These results demonstrated that Notch signaling is important in directing macrophage function in wound repair and provided a translational target for the treatment of non-healing diabetic wounds.

In the diabetic group of the current study, there was delayed wound healing evidenced by a significant increase in wound’s mean surface area, a significant decrease in epidermal thickness, and impaired angiogenesis as compared with the control groups. These results were accompanied by a highly significant increase in gene expression of Notch-1 signaling pathway, as there was pathological over-activation of notch1 and its downstream genes, combined with a significant decrease in gene expression of VEGF, SDF-1, and EPSCm (ß Integrin). These results were further confirmed by significant decreased CD31 immune-expression indicating defective new blood vessel formation and by a significant decrease in PCNA immune-expression indicating decreased EPSC proliferation. So, these results suggested a crucial role of pathological upregulation of Notch signaling in both diabetic EPSCs and diabetic endothelial progenitor cell dysfunction.

Many researchers demonstrated pathological activation of Notch 1 signaling in various cells essential for wound healing under diabetic conditions like EPSCs, keratinocytes, dermal fibroblasts, and dermal microvascular endothelial cells. When these cells were exposed to high glucose levels, the Notch1 signaling increased as reflected by increased expression levels of its target Hey1. In addition, pathological activation of Notch1 by high glucose levels resulted in negative effects on the migration of keratinocytes and fibroblasts as well as on the tube formation of vascular endothelial cells, all these processes are considered crucial for wound healing [8, 46].

Miloudi et al. 2019 [47] reported that chronic hyperglycemia-induced Notch 1 over-activation aggravated the rapid destabilization of the vascular endothelial cells leading to phosphorylation of VEGFR2 with increased production of NO. This in turn resulted in dissociation of the vascular endothelial cadherin/β-catenin complex. So, the high levels of reactive oxygen species induced an endothelial progenitor cells (EPCs) deficit leading to impairment of their abilities of angiogenesis, proliferation, differentiation, migration, and adhesion.

The expression of SDF-1, a vital factor for the recruitment of EPCs, is negatively controlled by Notch signaling. This provides a mechanism for how angiogenesis is regulated via Notch signaling. Interestingly, our results showed a significant inverse correlation between Notch expression and both VEGF and SDF-1 expression. In diabetic wound healing, pathologically activated Notch pathway impairs EPCs incorporation into the wound site secondary to decreased SDF-1 expression. This leads to a significant defect that contributes to impaired wound healing in diabetes Caiado et al. 2007 [48]. In addition, chronic hyperglycemia leads to upregulation of DII4, activating both canonical and rapid non-canonical Notch1 pathways. Subsequently, a hyperglycemia-induced Dll4–Notch1-positive feedback loop has been recognized to contribute to pathogenic sustained Notch activation in diabetes [49]. This is in concordance with the negative impact of Dll4-dependent Notch1 signaling on angiogenesis [8].

Therefore, Notch inhibition in diabetic wounds leads to improvement in EPSCs proliferation as well angiogenesis via facilitating the recruitment of EPCs. These results reflected the profound consequences of an increased Notch signaling for diabetic wounds [50]. Subsequently, Notch1 signaling blockage both in vitro as well as in vivo through either genetic or pharmacological methods was found to enhance wound healing in diabetes, via numerous mechanisms central for wound healing as cellular proliferation, migration, and angiogenesis. This signifies that Notch1 signaling is a new potential therapeutic target for the diabetic wound [51–53].

Recently, combined stem cell therapy, biomaterials, and/or autologous growth factors have been widely applied in fields of tissue regeneration [54–57], wound repair [58], regenerative plastic surgery [59], breast reconstruction [60–63], scalp reconstruction [64], complex abdominal wall repair [65], healing of venous and arterial ulcers 30999579, and hair growth [66–69].

Interestingly, recent studies have demonstrated that cell therapy and growth factors enhance diabetic wound healing. These researches suggested that MSCs have the ability to differentiate into other different cell types within the injured tissue to stimulate repair and regeneration of the skin. Also, PRP may provide a suitable microenvironment for MSCs to enhance proliferation and differentiation [9]. Indeed, growth factors present in the PRP play a fundamental role in enhancing safe and natural tissue healing encouraging its clinical application in chronic ulcers, soft tissue defect [70], and growth repair [71]. Scioli et al. reported that PRP combined with insulin enhances the chondro-/osteogenic differentiation of human adipose-derived stem cells suggesting their potential translational application in the field of regenerative medicine [72]. Moreover, the use of stromal vascular cell fraction-enhanced autologous fat grafts mixed with PRP improved adipose tissue maintenance and survival with better efficacy in the treatment of patients with burns sequelae and post-traumatic scars [73]. Additionally, combined hyaluronic acid and PRP reduced cost, patient pain, and healing period in chronic ulcer [74] and showed more efficacy in lower-extremity complex wounds [75] and in hidradenitis suppurativa [76]. Interestingly, many clinical studies reported the safety and efficacy of adipose tissue MSCs, PRP, and biomaterials on chronic wounds and soft tissue defects [77]. PRP injection showed also therapeutic efficacy in patients with androgenetic alopecia [78–80] and hair loss [81, 82]. Indeed, PRP treatment increased the mean number of hairs, total hair density, epidermal thickness and the number of hair follicles [83]. Further, combined PRP and human follicle MSCs positively influenced hair growth, stimulated hair follicle development, and suppressed apoptosis [84]. Of note, Gentile et al. provided an evidence of therapeutic positive outcomes of autologous stem cell-based therapy including PRP, human follicle stem cells, and adipose-derived stem cells in wound healing and hair regrowth [85]. PRP was also applied in oral and maxillofacial surgery [86]. PRP mixed with fat grafting improved breast reconstruction in patients with breast soft-tissue defects [87]. Therefore, in the current study, diabetic wounds were treated with adipose-derived MSCs (ADSCs) and PRP both individually and in combination to compare their therapeutic efficacy in diabetic wound healing and to assess their relation to Notch signaling pathway.

The current study revealed that Notch1 pathway-related genes were significantly downregulated to near the normal levels in ADSCs plus PRP group compared to the other treated groups and the diabetic group. There was improved wound healing with a decline in wound’s mean surface area beside the increase in all other parameters (area percentage of collagen, epidermal thickness, and CD 31-positive capillaries) in all treated groups, in comparison to the diabetic groups with significant amelioration detected in the ADSCs plus PRP group. Furthermore, the EPSCs were increased in all treated groups confirmed by gene expression of EPSC marker (ß Integrin) and PCNA immune-expression in the epidermal layer with significant improvement being observed in the ADSCs plus PRP in comparison to all treated groups and the diabetic group. Concomitant with these results, Motegi and Ishikawa 2017 [88] revealed that intradermal or intravenous administration of MSC-enriched cutaneous wound healing of acute and chronic skin injuries, for example, diabetic ulcers, acute excisional and incisional wounds, radiation ulcers, and burns in animals and humans. In animal models, the exogenous application of MSCs by topical and/or subcutaneous injection into incisional full-thickness wounds in normal or diabetic animals revealed speeding up of wound healing associated with increased reepithelization, angiogenesis, and decreased inflammation in the wounds [89]. Sorrell and Caplan 2010 [90] suggested two core mechanisms of wound healing acceleration by MSCs: (I) paracrine communication with resident cells in the wounds, infiltrating inflammatory cells, and antigen-presenting cells through the release of cytokines, growth factors, and ECM; and (II) their differentiation into resident EPSCs. These functions of MSCs can enhance reepithelization, angiogenesis, granulation tissue formation, ECM remodeling, and inhibit inflammation in wounds. Interestingly, PRP has been reported to enhance angiogenic effect and improve neovascularization of adipose-derived stem cells in ischemic hindlimbs [91]. Moreover, combined umbilical cord blood-derived (UCB)-MSCs and platelet-rich plasma (PRP) treatment showed improved regeneration and enhanced angiogenesis in combined radiation and wound injury model [92].

Additionally, results of the current study regarding angiogenesis clearly suggest that the ADSC and PRP therapy could induce a strong angiogenic effect in wound healing as CD31 immuno-expression as well as the gene expression of VEGF &STDF-1 were significantly increased in all treated groups with better results in ADSCs plus PRP group in comparison with diabetic wound group. This angiogenic response is critical for wound healing as it is necessary to increase oxygen and nutrient supply and to ensure the survival of keratinocytes. Moreover, it helps in sustaining the newly formed granulation tissue composed of a large number of the blood vessels, which were typically functioning and contained red blood corpuscles. The SDF-1 secreted from MSCs induces the survival of vascular endothelial cells, promotes vascular branching, and pericyte recruitment. These paracrine effects of MSCs play essential roles in angiogenesis rather than their direct differentiation to endothelial cells and/or pericytes [89]. In addition, some studies have revealed that angiogenic factors like VEGF, angiopoietins, and hepatic growth factor, which are released from injured tissue assist the recruitment of MSCs to the wound site [93]. In line with these results, Li et al. 2006 [94] stated that MSCs might differentiate into mature endothelial cells resulting in new blood vessel formation as well as expression of angiogenic growth factors through paracrine matter stimulating the neovascularization at the site of the wound. These results were explained by Volarevic et al. 2010, who stated that MSC administration leads to the secretion of some growth factors as well as some cytokines, such as PDGF, VEGF, bFGF, and angiopoietin-1 leading to enhancement of angiogenesis and wound healing [95]. Moreover, Suh et al. [96] reported that in a mouse model, MSC-treated groups demonstrated an enhanced wound-closure rate due to recruitment of monocytes/macrophages in addition to improving the neovascularization. Recently, MSC transplantation has been reported to activate EPCs leading to wound healing acceleration in a diabetic wound model [97].

Xie et al. 2013 [98] suggested that Notch signaling controlled MSCs homing to the injured tissues via SDF-1a/CXCR4 axis. CXCR4, the specific chemokine receptor of SDF-1a, is expressed in early passage MSCs and plays a pivotal role in the process of MSCs homing. Interestingly, Williams et al. [99] found that in endothelial cells, Notch signal activation induced by Dll4 downregulates CXCR4 expression and therefore inhibits responses toward SDF-1. This is the first report linking the Notch signaling pathway to CXCR4 regulation. Blocking Notch signaling may enhance MSC migration partly by upregulating the CXCR4 expression in MSCs. However, a significant barrier to the effective implementation of MSC therapy is the efficient engraftment of these cells into the injured tissues through interfering with Notch-CXCR4 signaling, which should significantly reduce the number of cells required to achieve therapeutic effects, and presumably provide better outcomes for patients. Moreover, Notch signaling plays an important role in MSC differentiation. It had been shown that the Notch signaling pathway controls the mesenchymal progenitor cell proliferation and differentiation throughout skeletal development [100]. Besides, Notch signaling preserves bone marrow mesenchymal progenitors via suppressing the osteoblast differentiation in the bone marrow [101]. In this context, Vujovic et al. [102] examined the crucial role of the Notch signaling pathway in human MSC proliferation and differentiation through using c-secretase inhibitors. They found that inhibiting c-secretase decreased the proliferation of human MSC, but it did not modify the expression of the osteoblast markers. This information suggested the essential role of the Notch signaling pathway in the regulation of MSC proliferation.

However, cell therapy alone can result in up to 20% of wounds remaining unhealed [103]. Researchers believe that this decreased efficacy may be due to the deficiency of numerous cytokines that encourage the migration and survival of EPSCs and EPCs. Additionally, owing to the inflammatory environment and the abnormal levels of growth factors in the wound area, the function of injected exogenous MSCs would be impaired due to the lack of stimulation by growth factors. The data of the current study coincides with these findings, supporting the hypothesis that MSCs in combination with PRP have a direct or indirect modifying effect on the angiogenesis via the growth factors as the PRP is a rich source of growth factors that stimulates angiogenesis in diabetic wounds improving the microenvironment for MSCs to work besides improving the niche of EPSCs. However, EPSCs and EPCs are insufficient alone for the treatment of diabetic wounds as Li et al. reported that EPCs, which can repair the damaged blood vessels and have angiogenetic abilities, are possibly suppressed due to the pathological activation of the Notch signaling pathway by chronic hyperglycemia [104].

In conclusion, the present study demonstrated the potential usefulness of combined ADMSCs and PRP in diabetic wounds. This could be attributed to modulating pathologically upregulated Notch 1 signaling, enhancing angiogenesis, and triggering epidermal cell proliferation and recruitment.

## Data Availability

Data are available on reasonable request from the corresponding author.

## Abbreviations

ADSCs: Adipose-derived mesenchymal stem cells
DLL4: Delta-like canonical Notch ligand 4
DFU: Diabetic foot ulceration
DM: Diabetes mellitus
EPSCs: Epidermal stem cells
Hes1: Hairy Enhancer of Split-1
Jag-1: Jagged-1
MSC: Mesenchymal stem cell
PRP: Platelet-rich plasma
SDF-1: stromal cell-derived factor 1
VEGF: Vascular endothelial growth factor
